# Clinical and imaging features of pediatric COVID-19

**DOI:** 10.1186/s13052-020-00917-1

**Published:** 2020-10-14

**Authors:** Yu Zhang, Ru-Ming Xie, Yu-Lin He, Li-Hong Xing, Li Dong, Jian-Zhong Zhang, Wei-Hong Xing, Xiao-Yan Lv, Yi-Bo Lu, Qiang Liu, Ling-Bo Lin, Gui-Zeng Liu, Li Li, Pan Li, Yuan-Zhong Xie, Zhi-Yu Ni, Xiao-Ping Yin, Hong-Jun Li, Bu-Lang Gao

**Affiliations:** 1 Affiliated Hospital of Hebei University, Baoding, 071000 Hebei Province China; 2 Beijing Ditan Hospital, Capital Medical University, Beijing, 100015 China; 3 The First Affiliated Hospital, NancHang University, Nanchang, 330006 China; 4 Baoding People’s Hospital, Baoding, 071000 China; 5 The Second Hospital of Xingtai City, Xingtai, 054001 China; 6 The Fifth Hospital of Shijiazhuang, Shijiazhuang, 050024 China; 7 The Fifth Medical Center of Chinese PLA General Hospital, Beijing, 100015 China; 8 The Fourth People’s Hospital of Nanning City, Nanning, 530023 Guangxi China; 9 Shandong Provincial Institute of Medical Imaging Research, Jinan, 250021 China; 10 Jinan Infectious Disease Hospital, Jinan, 250021 China; 11 Hebei Nanpi County Hospital, Nanpi, 061500 Hebei Province China; 12 Beijing Youan Hospital, Capital Medical University, No.8, Xi Tou Tiao, You An Men Wai, Feng Tai District, Beijing, 100069 China; 13 Jiangxi Province Jinxi County Hospital of Traditional Chinese Medicine, Jinxi County, 344800 Jiangxi Province China; 14 Medical Imaging Department, Shandong Province Tai’an City Central Hospital, Tai’an, 271000 Shandong Province China

**Keywords:** SARS-CoV-2, COVID-19, Novel coronavirus pneumonia, Children, Imaging, Epidemic

## Abstract

**Background:**

Pediatric COVID-19 is relatively mild and may vary from that in adults. This study was to investigate the epidemic, clinical, and imaging features of pediatric COVID-19 pneumonia for early diagnosis and treatment.

**Methods:**

Forty-one children infected with COVID-19 were analyzed in the epidemic, clinical and imaging data.

**Results:**

Among 30 children with mild COVID-19, seven had no symptoms, fifteen had low or mediate fever, and eight presented with cough, nasal congestion, diarrhea, headache, or fatigue. Among eleven children with moderate COVID-19, nine presented with low or mediate fever, accompanied with cough and runny nose, and two had no symptoms. Significantly (*P* < 0.05) more children had a greater rate of cough in moderate than in mild COVID-19. Thirty children with mild COVID-19 were negative in pulmonary CT imaging, whereas eleven children with moderate COVID-19 had pulmonary lesions, including ground glass opacity in ten (90.9%), patches of high density in six (54.5%), consolidation in three (27.3%), and enlarged bronchovascular bundles in seven (63.6%). The lesions were distributed along the bronchus in five patients (45.5%). The lymph nodes were enlarged in the pulmonary hilum in two patients (18.2%). The lesions were presented in the right upper lobe in two patients (18.1%), right middle lobe in one (9.1%), right lower lobe in six (54.5%), left upper lobe in five (45.5%), and left lower lobe in eight (72.7%).

**Conclusions:**

Children with COVID-19 have mild or moderate clinical and imaging presentations. A better understanding of the clinical and CT imaging helps ascertaining those with negative nucleic acid and reducing misdiagnosis rate for those with atypical and concealed symptoms.

## Background

The Coronavirus Disease-19 (COVID-19) pneumonia caused by the severe acute respiratory syndrome coronavirus-2 (SARS-CoV-2) which broke out in December, 2019, has now become a pandemic worldwide, affecting millions of people [1–7]. Since its breakout, the disease has been managed as one of class A infectious diseases. Class A infectious diseases indicate a class of diseases with the most infectiousness which will be treated using the most severe measures: detecting and managing the source of infection, cutting off the transmission pathway, and protecting the susceptible population. As the epidemic has entered its peak stage and better pathogen detection techniques have been developed, more children have been confirmed to be infected. Patients infected with this virus with or without symptoms were the primary source of spread through close contact and respiratory droplets as the major person-to-person transmission channels, and a family cluster is the main epidemic spread of COVID-19 in children [8]. The incubation period of infection of this virus is 1–14 days with most patients presenting with symptoms between 3–7 days [9, 10]. For children and teenagers infected with this virus, the age range was from newborns to 17 years with no clinical symptoms or with fever, fatigue, and dry cough, including accompanied upper respiratory symptoms like nasal congestion, runny nose, and sore throat [11–13]. Digestive symptoms may present initially like anorexia, nausea, vomiting, abdominal pain, and diarrhea. Pediatric COVID-19 infection is relatively mild in comparison to that of adults, and children with this disease have been reported to have a better prognosis with rare mortality [11, 14, 15]. A large proportion of infected children seems to be asymptomatic even though severe pediatric cases of COVID-19 have been reported [15]. However, if the infected child had some basic diseases, the infected COVID-19 might progress quickly to severe or critically severe type of disease requiring ICU admission and prolonged ventilation [16]. Fatal outcomes in pediatric COVID-19 pneumonia are rare, and up to June 2020, only a few deaths have been reported in children with COVID-19 [17, 18].

## Methods

This study was conducted to investigate the characteristics of COVID-19 infection in children so as to provide useful information for early diagnosis and treatment for children of this disease. This retrospective study was approved by the ethics committee of Affiliated Hospital of Hebe University with the reference number of 2020-TG-001, and the informed consent was obtained from the legal guardians of all participants. Between January and February 2020, children who had been infected with SARS-CoV-2 were enrolled. The inclusion criteria were children who had positive test of viral nucleic acid with or without pulmonary CT scanning since disease onset, and no other viral infection. The exclusion criteria were children with no infection of SARS-CoV-2. The clinical data were retrospective collected from electronic medical records, including age, sex, epidemic history, clinical symptoms, blood cell count, and imaging presentations of pulmonary lesions.

CT scanning was performed with three CT scanners: the LightSpeed VCT CT64 scanner (GE MEDICAL SYSTEMS, Tokyo, Japan), Siemens SOMATOM Perspective scanner (Shanghai, China) or Philips Ingenuity 64 scanner (Haifa, Israel). In CT scanning, the patient was put in the supine position, and the breath was held at the end of inhalation. The scanning covered the whole chest. The scanning parameters for the LightSpeed VCT CT64 scanner were tube voltage 120 kV, with the automatic milliampere technology (20–350 mA), noise index (NI) 18, pitch 0.984:1, matrix 512 × 512, slice thickness 5 mm, window width/level 1500/− 500 HU for the lung window, 350/40 HU for mediastinal window, and slice thickness 0.625–1.230 mm for reconstruction of the lung window in the axial position. The scanning parameters for the PHILIPS Ingenuity 64 row CT scanner were tube voltage 120 kV, with the automatic milliampere technology (50–300 mA), pitch 1, matrix 512 × 512, slice thickness 5 mm, window width/level 1500/− 550 HU for the lung window, 350/35 HU for mediastinal window, and slice thickness 1.0 mm for axial reconstruction of the lung window. The scanning parameters for the SOMATOM Perspective 64 CT scanner were detector collimation width 64 × 0.6 mm, tube voltage 120 kV, adaptive tube current (CARE Dose 4D), and high resolution algorithm reconstruction with the reconstruction slice thickness of 1.5 mm and slice interval 1.5 mm.

CT imaging analysis was performed by two imaging physicians independently. When in disagreement, a third physician would be involved to reach an agreement. The CT imaging presentations were analyzed with the following parameters: Disease distribution: right upper lobe, right middle lobe, right lower lobe, left upper lobe, left lower lobe; Lobes involved: one to five lobes involved, right, left and both lungs; Prevalence of lesion distribution: anterior portion, posterior, both anterior and posterior, peripheral, central, both peripheral and central areas; Within lungs: ground glass opacity, patchy high density lesion, consolidation, stripes, thickened bronchovascular bundles, along bronchus; Outside the lung: enlarged lymph nodes at the pulmonary hilum or inside the mediastinum, and pleural effusion. According to the line of axillary midline, the lung field in axial CT images was divided into anterior and posterior portion. The outer 1 / 3 of axial CT images was peripheral, and the rest was the central area.

According to the Guidelines for Diagnosis and Treatment of COVID-19 Infection by the China National Health Commission [10, 19], the COVID-19 was categorized into four types: mild with no or slight symptoms nor imaging presentations of pneumonia; moderate with fever, symptoms, and imaging manifestation of pneumonia; severe with any of the following: respiratory distress with a respiratory rate (RR) > 30 times/minutes, resting oxygen saturation less than 93%, or PaO2/FiO2 less than 300 mmHg (1 mmHg = 0.133 kPa); critically severe type with any of the following: respiratory failure requiring mechanical ventilation, shock, or combination with other organ failure requiring ICU intensive care. In radiological imaging diagnosis, COVID-19 was classified into early, progressive, severe and transforming stage [20].

### Statistical analysis

Statistical analysis was performed with the SPSS 20.0 software (IBM, Chicago, IL, USA). Qualitative data were presented as a rate or proportion ratio and tested with Chi Square test or exact probability test within a group. Quantitative data were presented as mean ± standard deviation (SD) and tested with paired t test if they followed normal distribution. If the quantitative data did not follow the normal distribution, they were expressed as median and interquartile range and tested with Chi Square test or exact probability test. Rank data were analyzed by rank sum test. The significant *P* value was set at < 0.05.

## Results

### Clinical features

#### Epidemical history

A total of 41 children with COVID-19 were enrolled including 23 males and 18 females with an age range of 0.5–14 years (mean 5.93) years (Table 1). In clinical classification, thirty patients were of mild (73.2%) type and eleven moderate (26.8%), and no children were of severe or critically severe types in this group. Thirty-three children were infected through family gathering, and eight had long been to the epidemic area within 2 weeks before admission.

**Table 1:** Clinical features of children with mild and moderate COVID-19

Clinical features	Mild(*n* = 30)		Moderate(*n* = 11)		X^2^	P
	No.	Rate(%)	No.	Rate(%)		
Age(y,mean ± SD)	6.40 ±4.31		4.67 ± 4.12		1.147	0.259
Sex (%) Boy	19	63.3	4	36.4	1.408	1.408
Girl	11	36.7	7	63.6		
Exposure to SARS-CoV-2					0.000	1.000
Family cluster	24	80.0	9	81.8		
Other exposure	6	20.0	2	18.2		
Fever	15	50.0	9	81.8	2.174	0.140
Low (37.2–38 °C)	8	53.3	3	33.3		
Mediate (38.1–39 °C)	5	33.3	5	55.6		
High (39.1–41 °C)	2	13.3	1	11.1		
Ultra-high (over41°C)	0	0.0	0	0.0		
Cough	3	10.0	5	45.5	4.382	0.036
Runny nose	3	10.0	1	9.1	0.000	1.000
Nasal congestion	1	3.3	0	0.0	-*	1.000
Eye pain	1	3.3	0	0.0	-*	1.000
Diarrhea	2	6.7	0	0.0	-*	1.000
Peripheral leukocyte						
normal	29	96.7	9	81.8	-*	0.170
abnormal	1	3.3	2	18.2		
Peripheral lymphocyte						
normal	23	76.7	7	63.6	0.191	0.662
abnormal	7	23.3	4	36.4		

Note: * Fisher exact probability test. Mild disease indicates the disease which has mild clinical symptoms, with no pneumonia shown on imaging. Moderate disease is characterized by fever, respiratory tract and other symptoms, with pneumonia shown on imaging

### Clinical symptoms

Among 30 children with mild COVID-19, seven presented with no clinical symptoms, fifteen had low or mediate fever as the primary symptom, and eight had cough, nasal congestion, diarrhea, headache, or fatigue. Among eleven children with moderate COVID-19, nine presented with low or mediate fever, accompanied with cough and runny nose, and two had no symptoms. Significantly (*P* < 0.05) more children had cough in moderate than mild COVID-19, but no significance existed in other data including age, sex, fever and other accompanied symptoms (Table 1).

#### Peripheral blood cells (Table 1)

Analysis of peripheral blood cells revealed normal peripheral leukocyte count in most children with mild (96.7%) and moderate (81.8%) diseases with no significant (*P* > 0.05) difference. The lymphocyte count decreased in 23.3% in mild and 36.4% in moderate COVID-19 with no significant (*P* > 0.05) difference.

#### CT imaging presentations

Thirty children with mild COVID-19 had no abnormal CT imaging in the lungs, and the rest eleven children with moderate COVID-19 had abnormal pulmonary CT imaging presentations.

#### Disease distribution

Pulmonary lesions were presented in the right upper lobe in two patients (18.1%), right middle lobe in one (9.1%), right lower lobe in six (54.5%), left upper lobe in five (45.5%), and left lower lobe in eight (72.7%).

Pulmonary lesions were presented in the upper lobe in five patients (45.5%), middle lobe in one (9.1%), and lower lobe in nine (81.8%) (Table 2). Most lesions existed in lower lobes followed by upper and middle lobes.

**Table 2:** CT imaging presentations of COVID-19 in children

CT imaging presentations	No.	%
Distribution in lobes		
Right upper lobe	2	18.1
Right middle lobe	1	9.1
Right lower lobe	6	54.5
Left upper lobe	5	45.5
Left lower lobe	8	72.7
Lobes involved		
One lobe	5	45.5
Two lobes	4	36.4
Three lobes	0	0.0
Four lobes	1	9.1
Five lobes	1	9.1
Only the right lung	1	9.1
Only the left lung	5	45.5
Both lungs	5	45.5
Distribution in the lungs		
Anterior	1	9.1
Posterior	8	72.7
Anterior and posterior	2	18.2
Peripheral	6	54.5
Central	2	18.2
Peripheral and central	3	27.3
Imaging presentations within lungs		
Ground glass opacity	10	90.9
Patchy high density	6	54.5
Consolidation	3	27.3
Enlarged bronchovascular bundles	7	63.6
Along bronchus	5	45.5
Imaging presentations outside the lung		
Enlarged mediastinal nodes	0	0.0
Enlarged hilum nodes	2	18.2
Pleural effusion	0	0.0

#### Lobes involved

Among 11 children with abnormal pulmonary CT imaging, one lobe was involved in five patients (45.5%), two lobes in four (36.4%), four lobes in one (9.1%) and five lobes in one (9.1%). No patients had the involvement of three lobes. Most patients had the involvement of one lobe followed by two and multiple lobes.

Only the right lung was involved in one patient (9.1%), only the left lung was involved in five (45.5%), and both lungs in five (45.5%). More patients had the involvement of bilateral lungs and left lung.

#### Prevalence of disease distribution (Table 2)

The disease was in the anterior portion of the lung in one patient (9.1%), in the posterior portion in eight (72.7%) and in both the anterior and posterior portions in two (18.2%), with the posterior portion as the most affected one.

The lesion was in the peripheral area in six patients (54.5%), central in two (18.2%) and both the peripheral and central areas in three (27.3%).

#### Imaging presentations within lungs

**Fig. 1:**
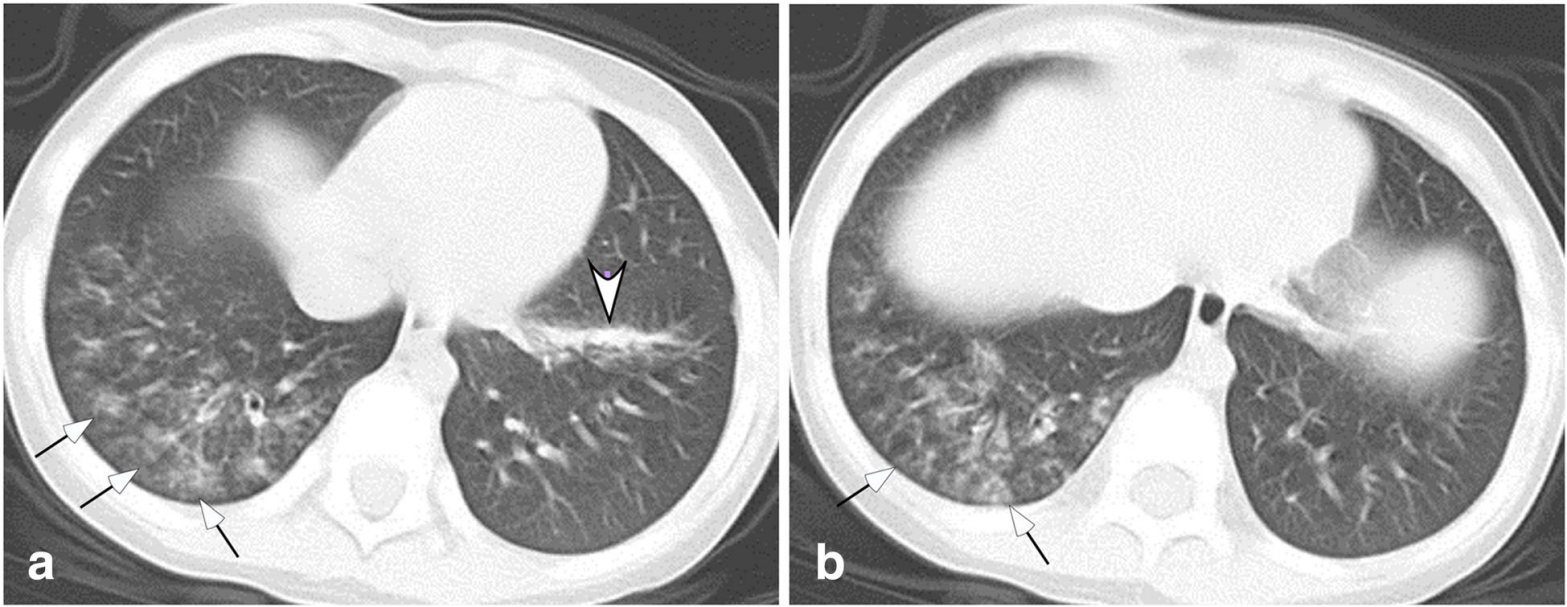
Moderate COCID-19 pneumonia in a 4-year-old boy who had fever and cough for 5 days before admission. **a** & **b**. Multiple lesions of ground glass opacity were presented in the right lower lobe (small arrows) along the bronchovascular bundle, and patchy lesions were present in the left lower lobe (arrow head, A) with partial consolidation

**Fig. 2:**
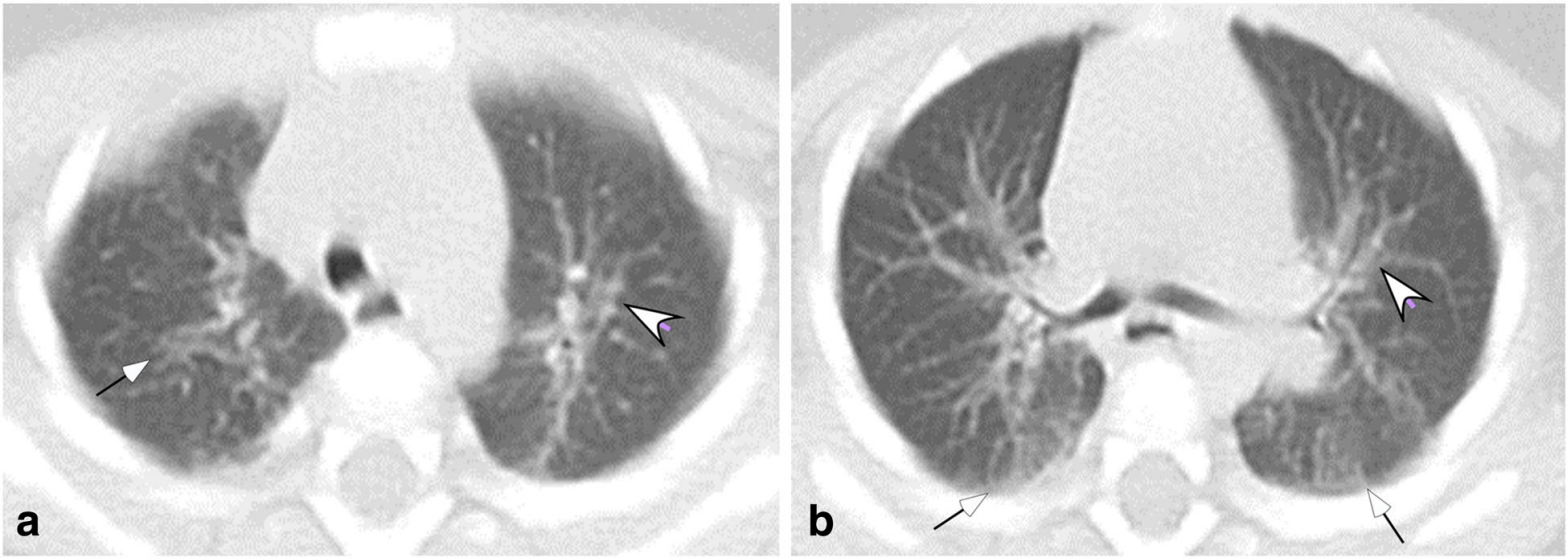
Moderate COVID-19 pneumonia in a 7-month-old girl who had fever and cough for 3 days before admission. **a** & **b**. The bronchovascular bundles were enlarged (arrow heads) in bilateral lungs with patches of ground glass opacity (small arrows) along the bronchovascular bundles

**Fig. 3:**
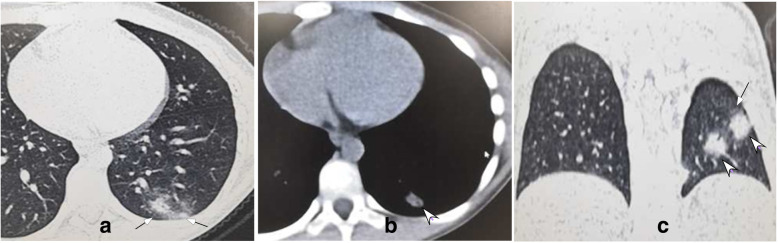
Moderate COVID-19 pneumonia in a 7-year-old girl with no symptoms. **a**. Patchy ground glass opacity (small arrows) was seen under the pleura in the left lower lobe. **b**. Partial consolidation was demonstrated in the center (arrow head). **c**. Patchy ground glass opacity (arrow) was shown in the left lower lung with consolidation in the center (arrow head)

The lesion was ground glass opacity in most patients and patchy high density in some patients along the bronchus with thickened bronchovascular bundles (Table 2 and Fig. 1, 2, 3). The lesion was ground glass opacity in ten patients (90.9%), patchy high density in six (54.5%), consolidation in three (27.3%), and enlarged bronchovascular bundles in seven (63.6%), with the lesions being located along the bronchus in five (45.5%).

#### Imaging presentations outside the lung

The lymph nodes were enlarged in the hilum of lung in two patients (18.2%) with no pleural effusion in none of the patients (Table 2).

#### Outcome

All children were recovered and discharged home. None of these patients suffered from pediatric inflammatory multisystem syndrome-temporally associated with SARS-CoV-2 (PIMS-TS) or multisystem inflammatory syndrome in children (MIS-C).

## Discussion

This study investigated the epidemic, clinical, and imaging presentations of all children who had been infected with the SARS-CoV-2 in our hospitals, and it was found that all children with COVID-19 were of mild and moderate type. The clinical and imaging presentations of children with infection of this virus are quite different from those reported in the epidemic center [15].

### Clinical and imaging presentations in children

The viral nucleic acid was positive in all 41 children including 30 mild children with negative pulmonary CT imaging, among which seven children had no clinical symptoms. Eleven children with moderate COVID-19 had abnormal pulmonary CT imaging. The pulmonary lesion was mostly restricted in a small area, with the lesion involving one lobe in 45.5% patients and more than one lobe in 54.5%. There were no severe or critically severe cases. The reasons for this are probably reduced toxicity of weakened viruses which had been transmitted many times among people in family gathering before infecting children [21], and during the viral transmission from people to people, the toxicity has been decreased and caused less damage to the body. Low immunity of children and under-developed cellular structure of the lung in children with different pulmonary receptors of angiotensin converting enzyme 2 (ACE-2) to bind the S protein of the SARS-CoV-2 viruses also play a role in decreased injury to the body [14].

In children, the predominant response to infectious stimulation is innate response to release higher levels of cytokines IL-6, IL-8, IL-10 and TNF-α to toll like-receptor stimulation [22]. Aging-related immune senescence in older patients predisposes these patients to elevated incidences and severity of pulmonary infection, and prolonged viral replication and delayed rise in cytokine levels have been reported in aged cotton rats compared to younger ones.. In healthy people, ACE-2 keeps homeostasis between angiotensin-2 (vasoconstriction, fibrosis, inflammation, and proliferation) and Ang-(1–7) pathways (vasodilatation, anti-fibrosis, anti-apoptotic, and anti-proliferation) [22–24]. SARS-CoV-2 viruses downregulate the ACE-2 expression and reduce the angiotensin-2 metabolism after infecting the pneumocytes. Increased angiotensin-2 elevates pulmonary vascular permeability and inflammation, consequently worsening the lung injury. In children, the underdeveloped lung cellular structure will probably reduce the role of SARS-CoV-2 virus in decreasing ACE-2 expression and reducing the angiotensin-2 metabolism. It has been proved that ACE-2 levels are decreased in old age and co-morbidities like hypertension and diabetes while the ACE-2 activity is increased in children, which probably explained worse pulmonary injury and prognosis in adults compared with children [22–24].

Although there have been very few severe cases of pediatric COVID-19, a syndrome of PIMS-TS or MIS-C has been reported in Europe and parts of North America [25–27]. In this syndrome, children infected with COVID-19 presented with systemic inflammatory responses, sharing some common characteristics with other pediatric inflammatory diseases including the toxic shock syndrome, Kawasaki disease, macrophage activation syndrome, and bacterial sepsis. Many of these children have myocardial dysfunction, coronary artery involvement, and gastrointestinal and systemic symptoms. This syndrome has been issued an alert from the Royal College of Pediatrics and Child Health and from the Centers for Disease Control and Prevention [26]. However, in our case series, there were no cases who suffered this syndrome. No other reports from China had presented such cases, either. The reason for this is not clear and is probably related to ethnicity.

### Different pulmonary imaging in children

The pulmonary lesion was mostly located in the external belt of the lung field under the pleura in multiple locations. In adults, the lesion was diffusely distributed in a larger area like reversed butterfly wings [2, 28–30], and in children, the lesion was relatively limited with rarely diffuse distribution. The lesion in children was small, with most presentations of ground glass opacity in patches but no presentations of “paving stone” signs or white lungs in severe cases. This kind of lesion may easily be misdiagnosed as ordinary bronchopneumonia without family gathering history. This distribution feature is in agreement with that of viral pneumonia which may readily invade the lung parenchyma around terminal bronchioles and respiratory bronchioles, with presentations of thickened walls of the bronchioles, centrolobular nodules and ground glass density nodules [31]. In adults, the lesion was mostly distributed in the external belt of the lung field under the pleura with rare distribution along the bronchovascular bundles [2, 28–30].

The lesions in our case series were distributed along the bronchovascular bundles and spreading from the central to the peripheral area, whereas pediatric cases in the serious epidemic area usually have the pulmonary lesion spreading from the external belt to the central area along the bronchovascular bundles [32]. In two patients in our series, the lymph nodes on the same or the contralateral side were enlarged while no lymph node enlargement has been reported in other pediatric series.

### Epidemic and clinical features

The incidence of COVID-19 is different in children in our study from that reported in other research [33]. In our study of multiple centers, the incidence of pediatric COVID-19 was 1.9% in five provinces in China. In a study investigating the incidence in 31 provinces in China with 1099 confirmed pediatric COVID-19 cases, the incidence was 0.9% [33]. The difference in the incidence might be caused by the cohort of patients, with ours being relatively small including only five provinces. The above incidences of pediatric COVID-19 in China were similar to those reported in other areas in the world [34–36]. The incidence of pediatric COVID-19 has been reported to be 1% of the total number of patients in Italy [34], 1.7% of the total number of recorded cases in the USA as of April, 2020 [35], and 4% of confirmed COVID-19 cases in Australia [36].

Children infected with SARS-CoV-2 have mostly been exposed to the epidemic focus or have definitive family gathering before disease onset. The first reported case of COVID-19 in a 10-year-old child without any symptoms in Shenzhen, China, was also caused by family gathering [8]. This indicated that close contact within the family is the primary spread manner of the SARS-CoV-2, similar to those of the SARS and MERS viruses [37].

In Italy, the incidence of transmission via obvious exposure to a family gathering was lower than that in other cohorts, which was probably caused by the late lockdown in Italy [34]. In this cohort of pediatric COVID-19 with a mean age of 3.3 years [34], 21% of the children were asymptomatic, 58% were of mild type, 19% had moderate disease, 1% were of severe type, and the remaining 1% were in a critical condition. In a multinational, multicenter cohort study in Europe [17], the mean age of the children with COVID-19 was 5.0 years with a male to female ratio of 1.15. In this study, 25% had pre-existing medical conditions, 62% were admitted to hospitals, 8% required ICU admission, 4% needed mechanical ventilation, 3% required inotropic support, and < 1% received extracorporeal membrane oxygenation. Our study had a mean age of 5.93 years in the children of COVID-19 including 73.2% in mild and 26.8% in moderate type, with no severe or critically severe type.

### Chest CT scans and lung ultrasound for children

In our study, the low-dose CT scans were used to investigate the pulmonary lesions of pediatric COVID-19. However, chest CT scans, although with diagnostic accuracy, have some shortcomings like high costs, high radiation exposure, and the need for sedation, which precludes routine use of CT scans for children [38]. CT scans should be reserved to compromised children needing admission to hospitals. In Europe, lung ultrasound is used more often than CT scans in stable children. In fact, both Chinese [39] and Italian [40, 41] researchers have provided the physical bases and lung ultrasound patterns in COVID-19 patients, indicating that lung ultrasound can be a useful tool to diagnose and monitor COVID-19 pneumonia. The study by Musolino et al. [42] further proved that routine use of lung ultrasound in assessment of children with suspected or confirmed COVID-19 is useful in diagnosing and monitering pediatric COVID-19 pneumonia, decreasing unnecessary radiation/sedation in children.

Some limitations existed in this study, including the retrospective nature, Chinese ethnicity enrolled only, and a small cohort of patients, which may potentially affect the conclusion of this study. A future study will have to resolve these issues for a better conclusion.

## Conclusions

In conclusion, children with COVID-19 have marked epidemic history with most patients having disease onset after family gathering. The clinical symptoms and pulmonary lesions were relatively mild or moderate. Children with COVID-19 have varied pulmonary CT imaging presentations, with the lesions primarily located in the lower lobes without diffuse distribution. For children who have had low-dose CT scan which is not typical for COVID-19, nucleic acid test result and epidemic history should be combined for a correct diagnosis. A better understanding of the pulmonary CT imaging presentations helps diagnosing COVID-19 so as to perform early isolation and intervention.

## Data Availability

The datasets used and/or analysed during the current study are available from the corresponding author on reasonable request.

## Abbreviations

COVID-19: Coronavirus Disease-19
SARS-CoV-2: Severe acute respiratory syndrome coronavirus-2
CT: Computed tomography
NI: Noise index
RR: Respiratory rate
SD: Standard deviation
